# Human Osteoblast Migration in DC Electrical Fields Depends on Store Operated Ca^2+^-Release and Is Correlated to Upregulation of Stretch-Activated TRPM7 Channels

**DOI:** 10.3389/fbioe.2019.00422

**Published:** 2019-12-12

**Authors:** Marco Rohde, Josefin Ziebart, Timo Kirschstein, Tina Sellmann, Katrin Porath, Friederike Kühl, Bachir Delenda, Christian Bahls, Ursula van Rienen, Rainer Bader, Rüdiger Köhling

**Affiliations:** ^1^Rostock University Medical Center, Oscar-Langendorff-Institute of Physiology, Rostock, Germany; ^2^Biomechanics and Implant Research Lab, Department of Orthopedics, Rostock University Medical Center, Rostock, Germany; ^3^Faculty of Computer Science and Electrical Engineering, Institute of General Electrical Engineering, University of Rostock, Rostock, Germany; ^4^Interdisciplinary Faculty, University of Rostock, Rostock, Germany

**Keywords:** galvanotaxis, osteoblasts, cell migration, TRP channels, bone regeneration

## Abstract

Fracture healing and bone regeneration, particularly in the elderly, remains a challenge. There is an ongoing search for methods to activate osteoblasts, and the application of electrical fields is an attractive approach in this context. Although it is known that such electromagnetic fields lead to osteoblast migration and foster mesenchymal osteogenic differentiation, so far the mechanisms of osteoblast activation remain unclear. Possible mechanisms could rely on changes in Ca^2+^-influx via ion channels, as these are known to modulate osteoblast activity, e.g., via voltage-sensitive, stretch-sensitive, transient-receptor-potential (TRP) channels, or store-operated release. In the present *in vitro* study, we explored whether electrical fields are able to modulate the expression of voltage-sensitive calcium channels as well as TRP channels in primary human osteoblast cell lines. We show migration speed is significantly increased in stimulated osteoblasts (6.4 ± 2.1 μm/h stimulated, 3.6 ± 1.1 μm/h control), and directed toward the anode. However, within a range of 154–445 V/m, field strength did not correlate with migration velocity. Neither was there a correlation between electric field and voltage-gated calcium channel (Ca_v_3.2 and Ca_v_1.4) expression. However, the expression of TRPM7 significantly correlated positively to electric field strength. TRPM7 channel blockade using NS8593, in turn, did not significantly alter migration speed, nor did blockade of Ca_v_3.2 and Ca_v_1.4 channels using Ni^+^ or verapamil, respectively, while a general Ca^2+^-influx block using Mg^2+^ accelerated migration. Stimulating store-operated Ca^2+^-release significantly reduced migration speed, while blocking IP3 had only a minor effect (at low and high concentrations of 2-APB, respectively). We conclude that (i) store operated channels negatively modulate migration speed and that (ii) the upregulation of TRPM7 might constitute a compensatory mechanism-which might explain how increasing expression levels at increasing field strengths result in constant migration speeds.

## Introduction

Fracture healing and bone regeneration, particularly in the elderly, remains a challenge, as healing is delayed and infringed by e.g., a reduction of the initial inflammatory phase and a reduced reaction, i.e., osteogenic differentiation, of mesenchymal stem cells (Gibon et al., 2016). Hence, there is an ongoing search for methods to activate osteoblasts. The application of electrical fields, first explored in the 1970s, is an attractive approach in this context (Brighton et al., 1985; Kaivosoja et al., 2015; Hiemer et al., 2016). Although it is known that such electromagnetic fields can lead to osteoblast migration (Ferrier et al., 1986), foster mesenchymal osteogenic differentiation (Hronik-Tupaj et al., 2011; Hiemer et al., 2016), and have also been reported to be beneficial in view of osteoporosis prevention (Wang et al., 2016), the data base on osteoblast migration is fairly narrow (published data refer to only a few dozen cells observed), and-perhaps more importantly-the mechanisms of osteoblast activation remain unclear.

One candidate mechanism for osteoblast activation could rely on a modulation of Ca^2+^-influx via ion channels, as this is known to alter osteoblast activity and helps bone formation (Thompson et al., 2012). Among these, both voltage-sensitive (Li et al., 2003), as well as stretch-sensitive, transient-receptor-potential (TRP) channels (Abed et al., 2009; Liu et al., 2014, 2015) are possible candidates. Specifically, both L-type, Ca_v_1.2, Ca_v_1.3 and T-type, Ca_v_3.2, voltage sensitive calcium channels are known to be expressed in osteoblasts (Shao et al., 2005; Tang et al., 2019), with Ca_v_1.2 (solely expressed in osteoblasts, and not osteocytes) probably mediating gap-junction-carried, interacellular calcium waves (Jorgensen et al., 2003). Importantly, this may have far-fetching clinical implications, as many elderly people, whose fractures often heal relatively slowly physiologically, are also treated for arterial hypertension with dihydropyridines: The latter drug class has been shown to infringe fracture healing (Moraes et al., 2011), and to promote osteoporosis (Kang et al., 2013). Ca_v_3.2 channels, in turn, are also expressed in osteocytes, where they are probably responsible for mechanosensing (Brown et al., 2016). In osteoblasts, however, shear-stress is apparently mainly communicated and sensed by TRPM7 (Roy et al., 2014; Liu et al., 2015), although in the past, other channels of the TRP family had been debated as well, such as TRPM4 (Thompson et al., 2012).

While for general osteoblast activation, higher Ca^2+^-levels are thought to be important, the matter is probably more complicated for migration. As data from other cell types (endothelia, fibroblasts, epithelial, malignoma cells) suggest, the interplay between TRP channels and store-operated Ca^2+^-release may determine migration. Specifically, Ca^2+^-oscillations at generally low baseline Ca^2+^ levels are thought to induce myosin-actin interactions at the cell front, and high Ca^2+^-concentrations at the opposing end of the cell likely lead to formation of focal adhesion-complexes (Tsai et al., 2015). Whether this also holds for osteoblast migration is as yet unclear.

In view of the above findings, highlighting the beneficial effects of electrical fields on the one hand, and the involvement of Ca^2+^-permeable channels for bone formation on the other, we set out to experimentally combine both of these issues and investigated whether (i) electrical fields are able to influence osteoblast migration, (ii) in which way this correlates to expression changes and function of voltage-sensitive calcium as well as TRP channels, and (iii) whether store-operated channels influence this galvanotactic migration in primary human osteoblast cell lines *in vitro*.

## Materials and Methods

### Cell Culture

To assess osteoblast reactions on electrical stimulation, we isolated human osteoblasts from femoral heads of patients (*n* = 14) undergoing a total hip replacement after obtaining patient's agreement and approval of the Local Ethical Committee (A 2010-10). Osteoblasts were isolated from cancellous bone as previously described (Lochner et al., 2011). Isolated cells were cultured in Dulbecco's Modified Eagle Medium (Pan Biotech, Aidenbach, Germany) supplemented with 10% fetal calf serum, 1% amphotericin B, 1% penicillin-streptomycin and 1% hepes-buffer under standard cell culture conditions (5% CO_2_ and 37°C). Ascorbic acid (50 μg/ml), β-glycerophosphate (10 mM), and dexamethasone (100 nM) (Sigma Aldrich, St. Louis, MO, US) were added to cell culture medium to maintain osteoblast phenotype. For cell migration experiments cells in passage three were used.

### Direct Current (DC) Stimulation Chamber and Experimental Procedure

**Figure 1:**
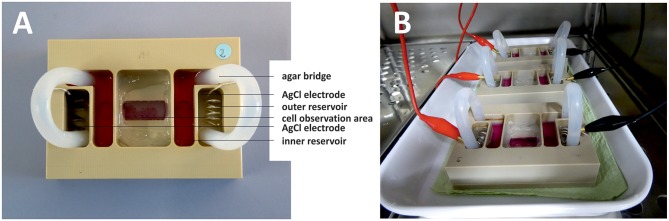
DC stimulation chamber. Photographs from top **(A)** and side **(B)** views. **(A)** Silver/silver chloride electrodes are positioned in outer reservoirs filled with Ringer's solution. Inner reservoirs are opening to the cell observation area and are filled with cell culture medium. Agar bridges ensure current flow between the electrodes while shielding cells from electrochemical processes occurring at the electrodes. **(B)** Three DC stimulation chambers inside the incubator. DC-stimulation is applied to the electrodes via crocodile clamps connected to a function generator outside the incubator.

**Figure 2:**
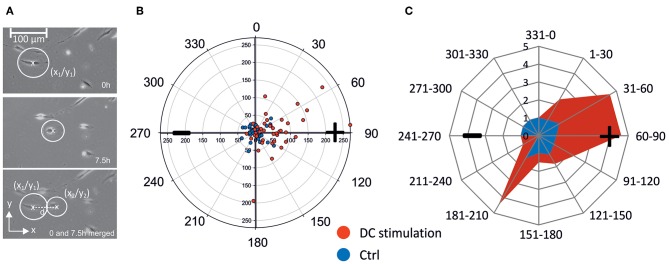
Calculation of cell migration. **(A)** Cells were encircled considering all cell extensions. Circle center coordinates were determined at the start and end points of the observation (in this case 0 and 7.5 h) and used for calculation of migration distance d. **(B)** Example of polar migration plot for *n* = 38 (control) and *n* = 41 (DC-stimulation) osteoblasts of one patient. Non-stimulated osteoblasts (CTRL) in this example remain largely within a 50 μm diameter and in all four quadrants, while >40% of stimulated osteoblasts (DC) migrate beyond 50 μm toward the anode mainly into quadrants 2 and 4 (Q2: 15 cells, Q4: 2 cells). **(C)** Example of angular migration plot. From the scatter polar plots such as the one shown in **(B)**, angular polar plots were constructed showing the contour of the migration. For this, the angle of migration from the original position (0–360°) was ascribed to a sector ranging from 1 to 10 and depicted as principal rays. Depending on the migration distance each cell was assigned to a migration value ranging from 1 to 32. For each sector a migration value depicted by the cross connections was calculated by summing up the migration values of the cells in one sector and dividing by the number of cells in the sector. Migration values of a representative experiment are shown in blue (controls) and red (DC stimulated cells). + anode; –cathode.

To gauge migration of osteoblasts in electric fields, we constructed a stimulation chamber and applied direct-current (DC) stimulation (Figure 1A). For this, the DC stimulation chamber described by Yang et al. was modified for osteoblast experiments (Yang et al., 2014). The chamber was made from polyether ether ketone and consists of two parts. Chamber parts were cleaned with 70% ethanol, washed with a mild detergent and rinsed excessively with distilled water before steam sterilization. A 24 × 50 mm coverslip was positioned in a groove in the upper chamber part and edges sealed with silicon paste (Korasilone, Obermeier GmbH, Bad Berleburg, Germany). Upper and lower chamber parts were bolted by 12 screws to ensure tight contact and prevent leakage and chambers were exposed to UV light for sterilization. Coverslips were coated with rat tail collagen (Advanced Biomatrix, San Diego, CA, USA) by incubation of 50 μm/mL rat tail collagen diluted in sterile 0.1% acetic acid for 1 h. Then solution was aspired and coverslips were washed twice with phosphate buffered saline (Biochrom, Berlin, Germany) before cell seeding. 2 × 10^3^ osteoblasts were seeded per chamber and cell adherence was allowed for 30 min. Coverslips were washed twice with medium to remove non-adherent cells, the chambers were sealed with a top coverglass, and silicon paste and cells accommodated to chamber overnight. Silver/silver chloride electrodes were placed into outer reservoirs separated from cell area to avoid harmful electrochemical reactions. Current flow was conducted to cell chamber by agar bridges consisting of 2% agarose (TopVision agarose, ThermoScientific, Waltham, MA, US) in Ringer's solution (Braun, Melsungen, Germany). For agar bridges 120 mm long silicon tubes with an inner diameter of 5 mm were used. Assembled chambers are depicted in Figure 1B. Current was applied to electrodes for 7 h via crocodile clamps using a DC power supply (Standard Power Pack P25, Biometra, Göttingen, Germany). Voltage was measured directly at the borders of the cell area with a 24 mm distance in between with a multimeter (Voltcraft VC220, Conrad Electronic, Wollerau, Switzerland) and adjusted during the experiments to maintain constant electric field strength. We used electric field strengths ranging from 154 to 445 V/m to test for field strength-dependence of migration velocity (*n* = 14 experiments, 21–42 cells per group, *n* = 339 and *n* = 341 cells from stimulated and unstimulated groups, respectively). Since electric field strength did not correlate with migration velocity within the range described above, we pooled all experiments on migration direction and velocity. In experiments determining channel expression changes, we likewise used electric field strengths from 131 to 441 V/m to determine whether expression changes depended on field gradient (*n* = 15, 23, and 24 experiments, respectively for Ca_v_3.2, Ca_v_1.4, and TRP-M7). Photographs were taken at 8 fields of view evenly distributed over the cell area at least at 3 time points with a Leica DMI 6000 and LAS X software. Exact overlay of photos taken at the start and end point was ensured with the image software GIMP and photos exported for evaluation of cell migration in ImageJ. Approximately five cells per field of view were evaluated by encircling cells including all cell extensions and centering cells. Circle center coordinates were determined at the start (X1/Y1) and end point (X2/Y2) and migration distance calculated as follows:

To illustrate and quantify migration within the electric field, we defined migration distance d $=\sqrt{{\left(X1-X2\right)}^{2}+{\left(Y1-Y2\right)}^{2}}$, where (X1-X2) and (Y1-Y2) represent the cell movement in x- and y-direction, respectively (Figure 2A). For each cell, we thus obtained a migration plot, which could be depicted in a polar coordinate system, plotting angle vs. migration distance in μm. A representative plot is shown in Figure 2B, which shows migration of osteoblasts under control conditions, and under DC-stimulation, from one donor; the anode is located at 90° angle. For better comparison of all experiments, we binned the migration angles in 12 sectors, and classified migration distances in a scoring system. Thus, the migration angle was calculated starting from the original cell position, and angles were assigned to the 12 sectors where sector 1–3 (1–90°) and 4–6 (91–180°) represent anode-directed migration, while sectors 7–9 and 10–12 (180–360/0°) represent cathode-directed cell migration. To construct star plots illustrating migration, migration distance of single cells was scored by assigning increasing distances to increasing values ranging from 1–32: 0–50 μm: 1; 51–100 μm: 2; 101–150 μm: 4; 151–200 μm: 8; 201–250 μm: 16; 251–300 μm: 32. Score values from all cells per sector were summed and divided by the number of cells per sector to obtain a migration value for unstimulated and DC stimulated cells for each cell experiment (Figure 2C). From these data mean migration values of all experiments were calculated for each sector.

### Reverse Transcription and Semiquantitative Real-Time Polymerase Chain Reaction (rtPCR)

To gauge the effect of DC stimulation on the expression of calcium-permeable channels, we conducted rtPCR analyses. For this, total RNA from cells was extracted using TRIzol reagent (Invitrogen Inc., USA) according to the manufacturer's instructions. 200 ng of total RNA were reverse-transcribed by adding random hexamers (3 μg/μl), dNTP mix (10 mM each; Invitrogen, Carlsbad, CA, USA), Moloney murine leukemia virus reverse transcriptase (200 U/μl) and RNasin plus RNase inhibitor (40 U/μl; both Promega Corporation, Madison, WI, USA). For real-time PCRs a mastermix containing 10x buffer, Mg^2+^ (4 mM), dNTPs (200 μM), Platinum taq polymerase (0.6 U/20 μl reaction, Invitrogen), and SYBR Green (concentration as recommended by manufacturer, Qiagen Inc., Valencia, CA, USA) were used. Primers (TIB Molbiol, Berlin, Germany) were added in a concentration of 20 μmol and are listed in Table 1. Real-time PCRs were performed using the ep mastercycler (software realplex 2.2, Eppendorf, Hamburg, Germany) with cycling parameters as follows: 95°C for 2 min once, followed by 95°C for 30 s and 59.2–62.5°C for 45 s, with normalized fluorescence read at 59.2–62.5°C (530 nm) for 40 cycles. To confirm single product amplification melting curve analysis and gel electrophoresis were done. Expression levels of Calcium-channel mRNA were normalized to GAPDH-housekeeping gene (Hs_GAPDH_1_SG, Qiagen) and analyzed by 2^−ΔΔct^ method. To assess changes in the expression of calcium-permeable channels, we first conducted explorative rtPCR on 31 channel types relative to GAPDH-expression to identify most prominently-expressed channels (Supplementary Figure 1). Of the 31 channels, only six were merely expressed at detection limit (Ca_v_1.1, Ca_v_2.3, TRPM3, TRPM1, and TRPP3). Taking an expression level of >1.75–10^−2^ relative to GAPDH as a cut-off level, three channel types were most prominently expressed: Ca_v_3.2, Ca_v_1.4, and TRP M7 (Supplementary Figure 1), whose primer sequences are listed in Table 1.

**Table 1:** Real-time PCR primer sequences.

**Gene**	**Forward primer**	**Reverse primer**	**bp**	**T**
Ca_v_1.4	TAAACATCAGAGGGGCAGGG	GGAAAGAAGTAGGCACAGCG	74	59, 2
Ca_v_3.2	ACAGTGGGTGCTCCGTAATG	CACAGGGAGAACTTCGGAGG	102	62, 5
TRPM7	TGTTTTCCTGCTTACCATGGGG	GGCTGAAATGTCCCATATCCCA	115	62, 5

### Pharmacological Tools to Assess Migration Mechanisms

To pharmacologically assess the functional contribution of TRPM7, of voltage-gated L-type and T-type Ca^2+^-channels and of store-operated channels as well as of IP3 receptors to migration speed modulation, we added the following substances to the medium: NS8593 as TRPM7 blocker (30 μM), verapamil as L-type Ca^2+^-channel blocker (100 μM), 2-Aminoethoxydiphenyl borate (2-APB) as store-operated channel opener at low concentrations and IP3 blocker at high ones (8 and 40 μM, respectively), all dissolved in 0.1% DMSO. The above drugs were obtained from Tocris Bioscience, Wiesbaden, Germany. Corresponding control experiments were done in 0.1% DMSO. To block T-type Ca^2+^-channels, channels, we used 50 μM Ni^2+^, and to block transmembraneous Ca^2+^-influx generally, Mg^2+^ (11.5 mM); in these cases, experiments with normal Mg^2+^-levels (2.3 mM) without DMSO served as controls.

### Statistical Analysis

Data were analyzed with SigmaPlot software (Systat, Erkrath, Germany) by testing for correlation or using the Mann-Whitney *U* test. *P* < 0.05 were considered statistically significant. DC experiments using different electric field strengths were repeated 14 times.

## Results

### Simulation of Electric Field Strength

**Figure 3:**
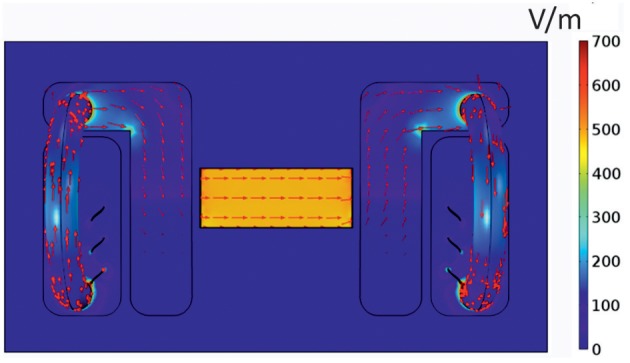
Current distribution in the electrotaxis chamber. Mathematical model of the distribution of the electric field amplitude |E| in V/m (depicted in colored scale) and the current density |J| in A/m^2^ (red arrows) inside the electrotaxis chamber. The applied voltage between the electrodes was assumed to be 129 V.

As electric fields strength and distribution/direction can importantly influence cell migration (Yang et al., 2016), we initially conducted *in silico* studies in order to obtain the electrical components inside the electrotaxis chamber presented in Figure 1. For this purpose, according to the DC stimulation, we computed the electric potential ϕ by solving Laplace's equation ∇ · (σ ∇ϕ) = 0 (resulting from Maxwell's equations in case of stationary currents) with the electric conductivity σ. For that, we used the FEM (Finite Element Method) software COMSOL Multiphysics^®^ version 5.1, in which we imported the 3D electro-taxis chamber after designing it in AutoCAD^®^ 2014. In Figure 3, modeling results for electric current density and the electric field distribution are shown.

From our simulation results, we can conclude that the electric field is homogenous inside the area where the cells are cultured. Thus, each single cell experiences the same electrical force-both in strength and direction.

### Migration of Human Osteoblasts

**Figure 4:**
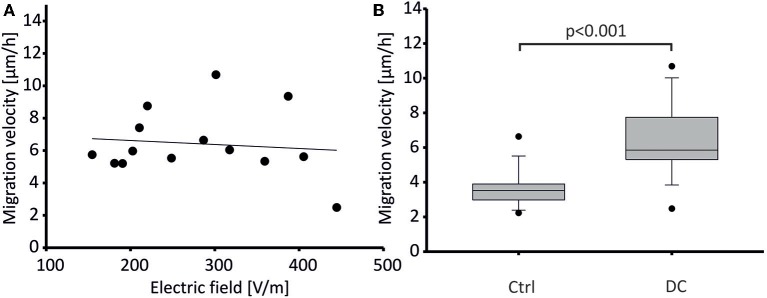
Migration velocity of DC-stimulated human osteoblasts. **(A)** Migration velocity of osteoblasts did not depend on electric field strength applied. Data points represent mean velocity values from 14 experiments. These were each obtained from *n* = 21–42 cells. Regression indicated as line. Regression coefficient: −0.0025, n.s. **(B)** Mean migration velocity (depicted as box plot showing median ± SD, and extreme values as dots) of osteoblasts stimulated by DC fields (DC) was significantly higher than that of unstimulated osteoblasts (Control). Data were obtained from *n* = 14 paired tissue samples each, *p* < 0.001.

Human osteoblasts were stimulated with constant direct current (DC) fields ranging from 150 to 450 V/m. In our experiments, migration velocity did not correlate with electric field strength (Figure 4A). Hence, mean migration velocity was calculated from all experiments (obtaining means from *n* = 21 cell chambers per group). Mean migration velocity in DC fields was 6.43 ± 2.06 μm/h (means ± SD of *n* = 21 cell chambers) while control cells without field application migrated with a significantly lower mean velocity of 3.6 ± 1.07 μm/h (means ± SD of 21 cell chambers, *p* < 0.001, Figure 4B). Calculating the average speed from the vector lengths of **Figure 6** rather than from cell chamber means, i.e., grouping by *n* = 14 donor cell cultures, we obtained similar average speeds of 6.96 ± 1.99 μm for stimulated cells, and 3.99 ± 1.09 μm for control cells, respectively (means ± SD, *n* = 14 each, *p* < 0.001, Mann-Whitney-Rank-Sum Test), showing that the difference persisted irrespective of the way we pooled the cells (chambers vs. donor cell culture).

**Figure 5:**
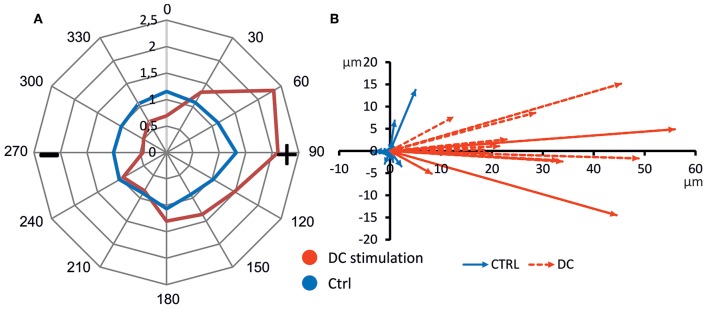
Directed cell migration toward anode. **(A)** Average polar plot of cell migration (*n* = 14 each). Cell migration was evaluated considering the migration angle determining the sector 1–10, migration distance assigned to a migration value ranging from 1 to 32 and number of cells per sector. Mean migration values of unstimulated and DC-stimulated cells from all experiments are presented, showing undirected cell migration of control, and anode-directed migration of DC-stimulated cells. **(B)** Mean migration vectors of all experiments (*n* = 14 each). Vectors were obtained by averaging migration vectors of all cells (*n* = 21–42) within one experiment (*n* = 14 each).

To gauge the directionality of migration, we constructed a cumulative star plot showing migration areas of all cells by calculating means of individual star plots (example shown in Figure 2C). As becomes evident from this plot (Figure 5), unstimulated osteoblasts practically all remain within a more or less even radius of 100 μm (migration value 1, Figure 5A). Stimulated cells (DC) cover a longitudinally-shaped area, directed toward the anode, and reach migration values of ~2.5, i.e., a mean migration distance of >150 μm (Figure 5A). In Supplementary Figure 2, all corresponding polar plots are shown.

**Figure 6:**
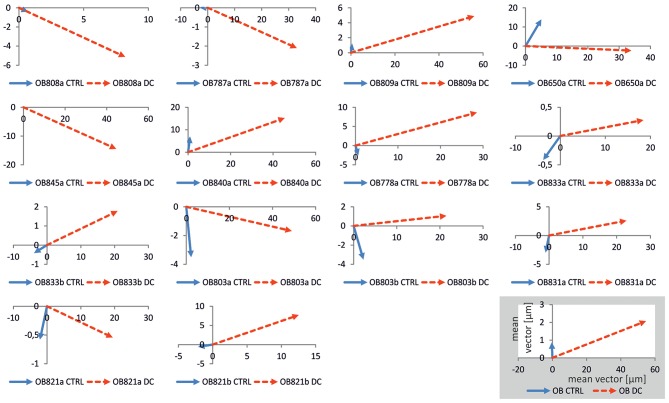
Plots of mean migration vectors for all experiments. Vectors were obtained by averaging migration vectors of all cells (*n* = 21–42) within one experiment. In all osteoblast cultures obtained from each donor (denoted as OB followed by number and suffix), vectors under control conditions (blue) were shorter than under DC-stimulation (red), and often orthogonal to, or opposing DC-stimulation vectors, the latter of which were all positioned in the right quadrants, i.e., in anodal direction. Distances in the coordinate systems are given in μm. In the inset, the calculated mean vectors for all experiments are given.

To further quantify migration, we obtained the main vector of all polar scatter plots shown in Supplementary Figure 2 by obtaining the mean of all vectors of individual osteoblasts (Figure 5B). Using this quantification, rather than showing the area of migration defined by the longest-migrating cells as in the polar plots, one obtains a weighted measure including cells which barely show any net migration at all. To ascertain that for each individual donor cell culture, DC-stimulation resulted in increased migration, we plotted corresponding vectors for all experiments. The resulting graphs are shown in Figure 6. As this shows, all vectors under DC-stimulation are larger than under non-stimulated, control conditions in all cases. Calculating all individual ratios of stimulated vs. unstimulated vector lengths, the mean ratio between stimulated and unstimulated vector length was 10.57 (range: 2.15–40.0; confidence interval of mean: ±6.1, 95% confidence level). More importantly, all vectors with stimulated osteoblasts were directed toward the anode, while in unstimulated osteoblasts, the mean vectors were roughly opposing this direction (in four cases), or roughly orthogonal (in eight cases). To quantify this further, we calculated the sine component of the vector (to obtain a relative value of directedness toward the anode). The median sine value for stimulated osteoblasts was 0.934, and for non-stimulated ones −0.063 (*p* < 0.001 Mann-Whitney-Rank Sum Test).

In conclusion, under DC-stimulation, human osteoblasts in our experiments clearly migrated to the anode, while non-stimulated osteoblasts moved randomly, and at lower speed.

### Calcium Channel Expression

Having confirmed anodal migration in osteoblasts, we hypothesized that changes in the expression of calcium-permeable channels might be correlated to migration. We tested this hypothesis by conducting rtPCR analyses of gene expression of those calcium-permeable channels whose basal expression in osteoblasts was most prominent. As mentioned in the Methods section, these were the voltage-sensitive channels Ca_v_1.4, Ca_v_3.2, and the stretch-activated channel TRP M7. While Ca_v_3.2 expression in osteoblasts was described in literature (Shao et al., 2005), the prominent expression of Ca_v_1.4 and TRP M7 is a novel finding.

**Figure 7:**
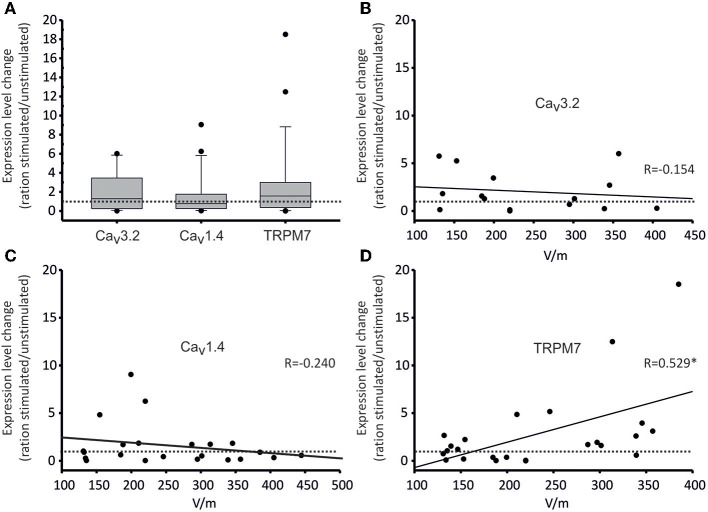
Gene expression of selected calcium channels under DC-stimulation. Gene expression changes are given as ratios of stimulated/non-stimulated cell cultures (as values on all ordinates). **(A)** Box plot of all expression ratios for Ca_v_3.2, Ca_v_1.4, and TRPM7 channels displaying 10, 25, 75, and 90 percentiles, as well as medians (Ca_v_3.2: 1.288, Ca_v_1.4: 0.761, TRPM7: 1.572) comparing stimulated/non-stimulated osteoblast cultures irrespective of stimulation intensity. The dotted line represents a ratio of 1, i.e., no change. Both Ca_v_3.2 and TRPM7 expressions were thus increased on average, while the expression of Ca_v_1.4 was decreased. Over the whole range of stimulation voltages, however, these changes were not significant. **(B–D)** Dot plots of correlations of expression ratios of Ca_v_3.2 **(B)**, Ca_v_1.4 **(C)** and TRPM7 **(D)** channels and voltages of DC-stimulation. Each dot represents one experiment. Again, the dotted line represents a ratio of 1, i.e., no change. Continuous lines show linear regressions; the corresponding regression coefficients R are given in the insets. For TRPM7, the channel expression increased significantly (^*^ denotes significant regression) with increasing stimulation strength (*R* = 0.529, *p* = 0.0079).

Regarding the effects of DC-stimulation, as can be seen in Figure 7, RNA expression of all channels changed with stimulation, albeit in different directions (Figure 7A). Only in one channel type (TRPM7), the change was significantly correlated to stimulation-intensity (Figure 7D). Specifically, regarding Ca_v_3.2, in the majority of experiments (9/15), there was an increased expression (up to 10 higher), while in 6/15 experiments, there as a decrease (down to 0.01; Figure 7B). These changes in expression were, however, not related to stimulation strength (Figure 7B). Regarding Ca_v_1.4, in 13/22 experiments, interestingly there was a down-regulation, whereas in 9/22, the expression was mildly upregulated (Figure 7C). These changes, again, did not correlate to stimulation strength (Figure 7C). Lastly, TRPM7 channel expression was upregulated in 15/24 experiments, and downregulated in 9/24 (Figure 7D). Importantly, the stimulation strength and expression increase were significantly correlated (*R* = 0.529; Pearson Correlation Coefficient *p* = 0.0079; Figure 7D). Thus, the higher the applied DC field, the higher was the TRPM7 expression compared to the respective control-while at the same time, field strength increase did not increase migration speed (cf. Figure 4), suggesting that TRPM/TRPM7 expression and migration speed are not likely to correlate directly.

### Migration Mechanisms

To unravel the link between channel expression changes and migration speed, and to obtain first hints on the mechanisms of migration under galvanotactic stimulation, we used pharmacological tools to assess the contribution of transmembraneous Ca^2+^-influx, and more specifically of TRPM7 and voltage-gated Ca^2+^-channels, to migratory activity. Likewise, we gauged the role of intracellular Ca^2+^ release, i.e., of store-operated Ca^2+^-release and IP3 receptor function.

**Figure 8:**
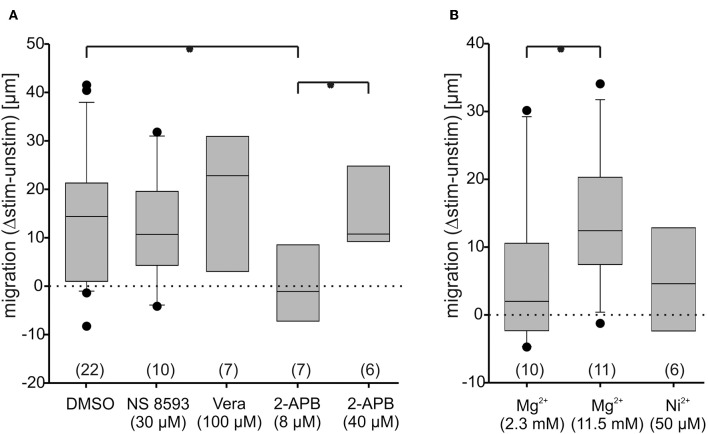
Osteoblast migration speed changes under blockade of transmembrane Ca^2+^-conductances and under activation of store-operated channels. Changes of anodal migration speed are given as differences between migration with and without DC-stimulation (Δstim.-unstim.), given as box plots. The dotted lines represent no net movement change. In a fist set of experiments **(A)**, the role of TRPM7 gating (blocked by NS 8593, 30 μM), of L-type Ca^2+^-channel ion flux (blocked by verapamil; Vera, 100 μM), of store operated channels (activated by 2-APB, 8 μM) and of endoplasmic reticulum IP3-receptor function (blocked by 2-APB at 40 μM) was compared to DMSO (solvent of the drugs at 0.1%) as control. In a second set **(B)**, the role of T-type Ca^2+^-channel ion flux (blocked by Ni^+^, 50 μM) and of transmembraneous Ca^2+^-flux as a whole (blocked by 11.5 mM Mg^2+^) was compared to conditions in normal extracellular Mg^2+^ concentration (2.3 mM). The number of experiments is given in brackets. Each experiment was conducted on 20–40 cells per group. ^*^Denote significant differences in Mann-Whitney *U*-test, with *p* < 0.05.

Blocking TRPM7 channel gating using NS8593, a compound which blocks gating of the TRPM7 channel, but not its kinase activity (Hofmann et al., 2014), did not significantly alter migration speed (Figure 8A; Supplementary Table 1), confirming that the link between TRPM7 expression and migration speed is not a direct one. Since Ca^2+^-flux through TRPM7 channels thus obviously plays a minor role at most, we checked whether transmembraneous flux via T-type channels (such as Ca_v_ 3.2) or L-type channels (such as Ca_v_ 1.4) might influence migration using Ni^2+^ (50 μM) and verapamil (100 μM) as blockers. In both cases, neither channel type turned out to be essential, since migration speed was unaltered with respect to control (Figures 8A,B; Supplementary Table 1), although transmembraneous Ca^2+^-influx as such was confirmed to be negatively correlated to migration speed, since high Mg^2+^ concentrations more than doubled the migration speed (Figure 8B; Supplementary Table 1). The channels mediating this influx, however, remain elusive at this point, although it is conceivable that indeed many if not all of transmembraneous ones (25 possible candidates are expressed as listed in Supplementary Figure 1) might contribute to some degree, which would at least be in line with the finding that blocking specific channels exclusively does not have a significant effect.

Since store-operated channels were reported to be pivotal for migration in other cells (Tsai et al., 2015), we next investigated their contribution. Using 2-APB at a low concentration (8 μM) know to open store operated channels (Prakriya and Lewis, 2001), migration was indeed virtually arrested (Figure 8A). Using the same compound at high concentrations (40 μM) – a condition in which presumably additionally IP3-receptors are blocked, and TRPM 1 and TRPM2 channels are opened (Prakriya and Lewis, 2001)-motility was restored to normal (Figure 8A), demonstrating the importance of IP3 receptor function.

In summary one can conclude that store operated channels negatively modulate migration speed, while transmembraneous Ca^2+^ flux is essential, but not carried by any one channel alone.

## Discussion

In the present study, our fist aim was to characterize galvanotactic migration in human osteoblasts, which we found to be anodal under DC-stimulation. Although galvanotactic migration has been reported in osteoblasts in earlier studies (Ferrier et al., 1986), the directions of migration in our study and the cited one are not in agreement, at least at first sight. Thus, Ferrier et al. (1986), in two out of three experiments with two different rat osteoblast cell lines, reported either no significant directed migration at a comparable field strength of 100 V/m, or, after extended exposure (>3 h), a cathodal movement with a rather variable mean velocity of 3- to 20 μm/h, shown by roughly 78% of all cells. In the cited paper, a tenfold increase of field strength (1 kV/m) resulted in increased migration velocity of ~130 μm/h. These findings do not match with those from the present study, since we found, on average, an anodal movement of cells at a speed of ~7 μm/h at electric field strengths between those described in the cited paper (100–450 V/m). The reasons for this discrepancy could be several: First, in the study by Ferrier et al. rat osteoblast cells lines from immature rats were used, while here we report data from primary human osteoblasts derived from adult persons. There is thus both a species and developmental difference. The latter may be of particular importance, since it has been shown that donor age qualitatively influences both responses to physical stimuli and proliferative signals of osteoblasts (Shiels et al., 2002; Evans et al., 2006). Supporting this ontogenetic argument, in the same study by Ferrier, interestingly more “mature” cells, i.e., osteocytes, actually do migrate into the opposite direction (Ferrier et al., 1986). It is thus conceivable that the age of the cells is underlying the differences seen. Moreover, the cited study is based only on 75 cells in total, and three experiments, such that we can expect variability to be quite higher than in the present paper.

As a methodological note, the migration direction was determined as net migration, i.e., the path the osteoblasts migrated along was usually not linear, but in most cases curvy, and often convoluted particularly in the control situation. Obviously, hence, all cells moved, at least to some degree, including osteoblasts under control conditions. Further, there was a large degree of variability among the cells even within one donor sample, both under control and under DC-stimulation conditions, as evidenced by the polar plots in Supplementary Figure 2. Hence, the migration plots (Figures 2, 5A) showing the area covered by the cells actually delineate the boundaries of migration defined by the most mobile cells in a batch. To obtain a measure of the average behavior of cells, the vector plots (Figures 5B, 6) give a more realistic picture of the movement directionality of the whole osteoblast population.

Our second aim after quantifying galvanotaxis in osteoblasts was to identify the possible sources of transmembraneous Ca^2+^ flux, which undisputedly is important for migration (Funk, 2015; Tsai et al., 2015). Hence, as a first step we analyzed the expression of different Ca^2+^-permeable transmembraneous channels and found 25 different types as shown in Supplementary Figure 1. To see Ca_v_3.2 and Ca_v_1.4 expressed is a novelty as such, since both have been so far only described in the retina and in immune cells (Striessnig et al., 2014; Sang et al., 2016). As for Ca_v_1.4, protein kinase A (PKA) renders this channel more Ca^2+^-permeable (Sang et al., 2016), and since activated PKA has been shown to increase bone growth (Tascau et al., 2016), there is a hypothetical link between Ca_v_1.4 and osteoblast activation. Likewise, TRPM7 has been linked to migration of at least non-osteoblast cells in a number of studies (Wei et al., 2009; Funk, 2015; Tsai et al., 2015). We were therefore interested in the dependence of channel expression on field strength, and chose to explore this correlation for the three most prominently expressed channels (Ca_v_3.2, Ca_v_1.4, and TRPM7). These experiments showed that only TRPM7 was upregulated stimulation intensity-dependently. Since migration speed did not change within the field strength range tested, there was, however, no positive correlation between TRPM7 up-regulation and migration speed.

To explore the role of TRPM7 further, we used pharmacological blockade of the gating/channel by applying NS 8593 and found that neither this channel, nor indeed L-type (blocked by verapamil) or T-type (blocked by Ni^2+^) voltage gated Ca^2+^-channels, play a singularly decisive role for migration-although transmembraneous Ca^2+^-flux as such, blocked by Mg^2+^ (at high concentrations of 11.5 mM which block virtually all flux) is pivotal in the sense that blocking it leads to an increase in migratory speed. The function of store-operated Ca^2+^-release, in turn, is also to slow down migration, since stimulating it at low doses of 2-APB froze migration practically completely. Also IP3 receptor function is essential, since blocking it under conditions of store-operated channel activation restores mobility. Hence, our data on osteoblasts are in agreement with data from other cell types such as fibroblasts, endothelial cells, blood cells and tumor cells, where high levels of endogenous Ca^2+^-release block migration, while low levels of Ca^2+^/Ca^2+^ influx, or specifically, flickers or waves of Ca^2+^ turnover from low baseline levels promote migration (Wei et al., 2009; Funk, 2015; Tsai et al., 2015).

Does TRPM7 have no function, even though it is upregulated field-strength dependently? As the high Mg^2+^-experiments show, transmembraneous Ca^2+^-flux does influence migration – when it is blocked by high concentrations of Mg^2+^, migration is accelerated. Hence, the Ca^2+^-waves/flickers necessary for myosin activation (Wei et al., 2009) must originate from internal stores while the transmembrane flux probably adjusts baseline level Ca^2+^-levels. Blocking just one of these channels (e.g., TRPM7) apparently leaves many others active the cells can resort to. Nevertheless, in many other cell types, including fibroblasts, TRPM7 were identified as being involved in migration-associated Ca^2+^ flux (Wei et al., 2009; Funk, 2015; Tsai et al., 2015).

In addition to providing transmembraneous Ca^2+^ flux, TRPM7 is supposed to be upregulating phosphoinositol3-kinse (PI3K; shown to be involved in fibroblast motility; Allen et al., 2013) and protein kinase B (PKB/AKT) pathways, and consecutively growth factor transcription (Zhang et al., 2017). Importantly, upregulation of TRPM7 (in the cited study by using platelet derived growth factor, PDGF) can increase osteoblast motility (Abed and Moreau, 2009). Even our negative results using the TRPM7 blocker NS 8593 do not rule out such a role in osteoblasts, since the blocker only influences gating / Ca^2+^ flux, but not another important property of the channel: its C-terminal serine/threonine protein kinase activity (Hofmann et al., 2014). This kinase activity endows it with a multiplicity of pathway modulation capabilities, including phosphorylation of tropomodulin 1, controlling actin filament dynamics (Visser et al., 2014), and an interaction with proteins involved in cytoskeletal organization and adhesion dynamics (Middelbeek et al., 2016), such as tropomodulin 1, α-actinin, myosin and p116Rip, an F-actin bundling protein (Mulder et al., 2003; Middelbeek et al., 2016). While this has only been shown for neuroblastoma cells up to now, osteoblast motility might likewise be influenced in the same manner. TRPM7 could thus act by parallel both, cation conductance and substrate phosphorylation. Further experiments are needed to elucidate the role of TRPM7 kinase activity.

Another open question is whether TRPM7 is actually gated under DC-stimulation. The channel is voltage independent, such that a direct impact of membrane potential can be ruled out (Visser et al., 2014) (besides the fact that DC-stimulation would not induce repetitive gating even in voltage-dependent channels). It is, however, mechanosensitive, and cell stretch activates it (Numata et al., 2007). At least in fibroblasts, electrical stimulation-induced migration has been shown to be depending on integrin functionality, and intracellular pathway signaling-in that instance membrane bound phospholipase C (PLC) which would result in inositol triphosphate (IP3)-induced intracellular calcium release (Sun and Cho, 2004). Future studies should explore this context in osteoblasts.

What could be the function of TRPM7 upregulation then? The observation that increasing field strengths-at least within the range of 150–450 V/m-did not lead to further increases in migration speed is in disagreement with the modeling study (Vanegas-Acosta et al., 2012), which predicted a linear correlation resulting in speeds of ~1.8 to ~4 μm/s (range 0–500 V/m). One reason for this discrepancy may be adhesion, which has to be overcome, a factor which would result in non-linear behavior. Another reason might be that mobility was rate-limited by factors such as the rate of actin conformational change, which at 21°C is only ~50% of maximum (Jacobs et al., 2011), and otherwise depends on intracellular Ca^2+^-levels, saturating at ~10 μM, at least in thin filaments (Sich et al., 2010). In view of the field-strength dependent up-regulation of TRPM7, another intriguing possibility would also be that TRPM7-upregulation might actually constitute a homeostatic mechanism to raise Ca^2+^ flux to limit migration speed-obviously, this hypothesis also awaits testing in further experiments.

## Data Availability Statement

The datasets generated for this study are available on request to the corresponding author.

## Author Contributions

RK, RB, and UR conventionalized the study. JZ, TS, KP, and FK conducted the cell migration experiments. BD, CB, and UR provided the modeling data. RB provided the human cell lines. MR, TK, and RK provided the analysis methods. TS, KP, and RK generated the illustrations. RK, UR, and RB wrote the paper.

### Conflict of Interest

The authors declare that the research was conducted in the absence of any commercial or financial relationships that could be construed as a potential conflict of interest.

## Supplementary Material

Supplementary Figure 1 Expression levels of candidate Ca^2+^-permeable channels in osteoblasts (abscissa) under control conditions. The bar chart shows ratios of expression levels relative to expression of housekeeping gene GAPDH (ordinate). Cut-off was chosen at values of 1.75 × 10^−2^, or larger. The red ellipses encircle the channels chose for further investigation.

Supplementary Figure 2 Polar migration plots of all experiments. These show migration of osteoblasts of individual donor cultures (denoted as OB followed by number and suffix) under control conditions (black dots), and under DC-stimulation (red dots). Each dot represents one osteoblast; coordinates are given in μm. In each case, the anode is located at 90° angle, i.e., in vicinity of the right-hand quadrants.

Supplementary Table 1 Osteoblast migration speed changes under blockade of transmembrane Ca^2+^-conductances and under activation of store-operated channels.
