# Supplementary figures and images for: Human Osteoblast Migration in DC Electrical Fields Depends on Store Operated Ca2+-Release and Is Correlated to Upregulation of Stretch-Activated TRPM7 Channels
Source: Front Bioeng Biotechnol. 2019 Dec 12;7:422. doi: 10.3389/fbioe.2019.00422 (PMC6920109; doi:10.3389/fbioe.2019.00422)

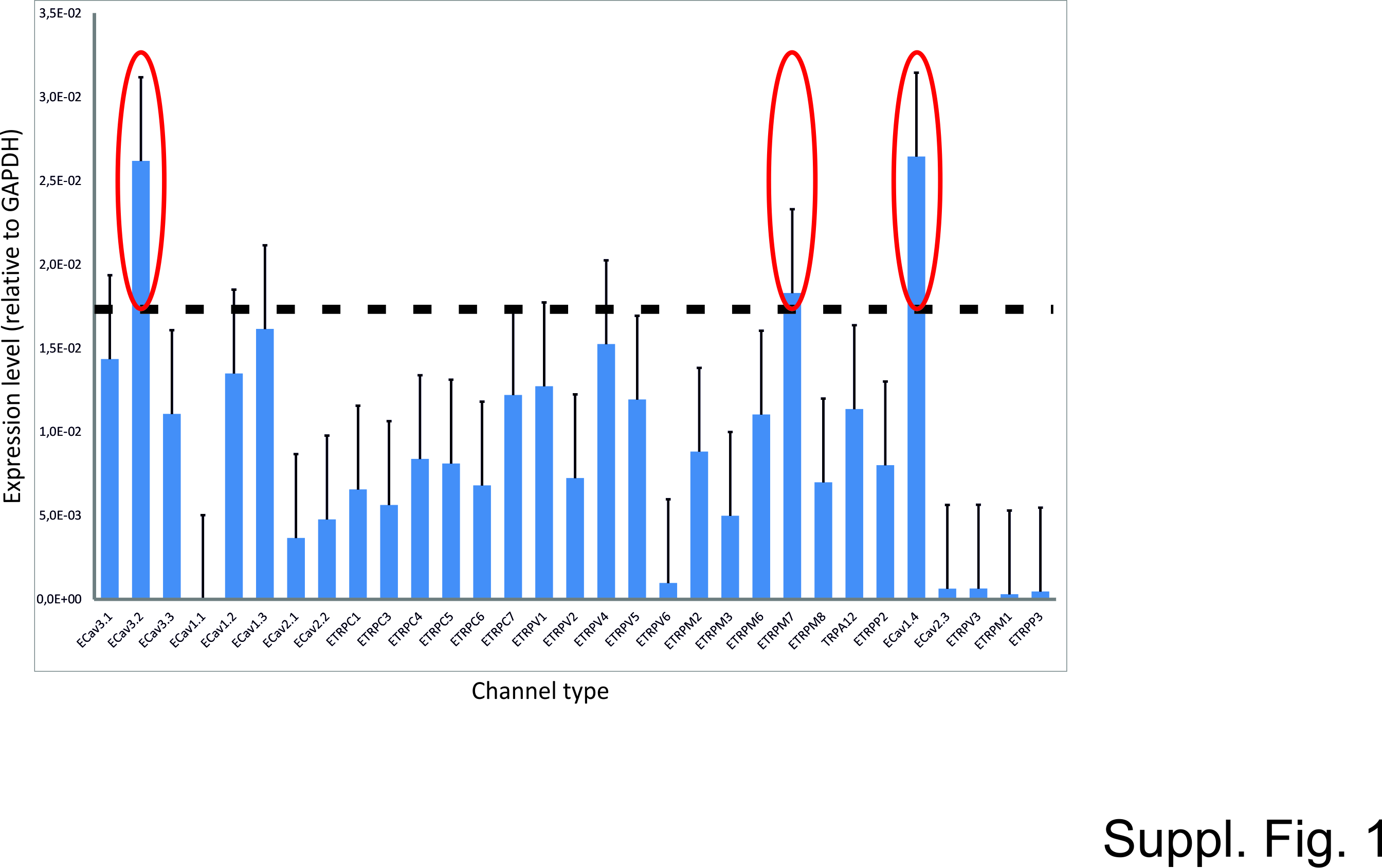

Supplement: Supplementary Figure 1 — Expression levels of candidate Ca2+-permeable channels in osteoblasts (abscissa) under control conditions. The bar chart shows ratios of expression levels relative to expression of housekeeping gene GAPDH (ordinate). Cut-off was chosen at values of 1.75 × 10^−2^, or larger. The red ellipses encircle the channels chose for further investigation. [file Image_1.JPEG]

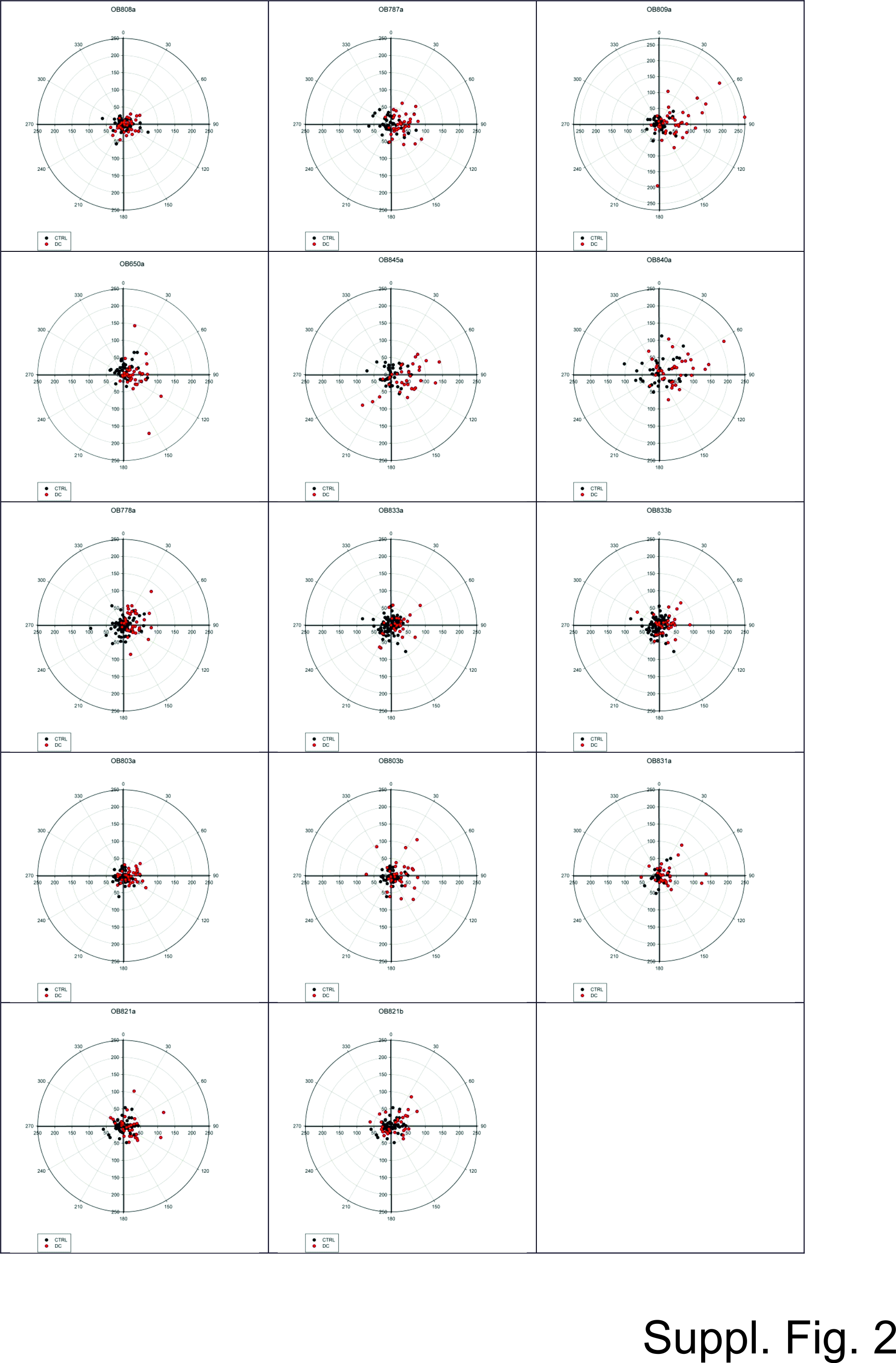

Supplement: Supplementary Figure 2 — Polar migration plots of all experiments. These show migration of osteoblasts of individual donor cultures (denoted as OB followed by number and suffix) under control conditions (black dots), and under DC-stimulation (red dots). Each dot represents one osteoblast; coordinates are given in μm. In each case, the anode is located at 90° angle, i.e., in vicinity of the right-hand quadrants. [file Image_2.JPEG]
