# Supplementary material for: Human Osteoblast Migration in DC Electrical Fields Depends on Store Operated Ca2+-Release and Is Correlated to Upregulation of Stretch-Activated TRPM7 Channels
Source: Front Bioeng Biotechnol. 2019 Dec 12;7:422. doi: 10.3389/fbioe.2019.00422 (PMC6920109; doi:10.3389/fbioe.2019.00422)
Supplement: Supplementary Table 1 — Osteoblast migration speed changes under blockade of transmembrane Ca2+-conductances and under activation of store-operated channels. [file Table_1.pdf]

Suppl. Table 1: Osteoblast migration speed changes under blockade of transmembrane Ca^{2+}-conductances and under activation of store-operated channels.

	**DMSO**	**NS8593**	**Verapamil** 100 µM	**2-APB** 8µM	**2-APB** 40 µM	**Mg^{2+}** 2.3 mM	**Mg^{2+}** 11.5 mM	**Ni^{+}** 50 µM
mean	13.42	11.80	18.44	1.93	15.97	5.68	14.47	5.52
SD	13.03	10.29	13.86	9.86	9.64	10.82	9.28	8.52
SEM	2.78	3.25	5.24	3.73	3.94	3.42	2.80	3.48
n	22	10	7	7	6	10	11	6

Mean net (stimulated-unstimulated) migration speed (µm), number of experiments (n), standard deviation (SD) and standard error of the mean (SEM) underlying box plots in Fig. 8.
